# Antioxidant Properties and Aldehyde Reactivity of PD-L1 Targeted Aryl-Pyrazolone Anticancer Agents

**DOI:** 10.3390/molecules27103316

**Published:** 2022-05-21

**Authors:** Natascha Leleu-Chavain, Romain Regnault, Hania Ahouari, Raphaël Le Biannic, Mostafa Kouach, Frédérique Klupsch, Romain Magnez, Hervé Vezin, Xavier Thuru, Christian Bailly, Jean-François Goossens, Régis Millet

**Affiliations:** 1 Univ. Lille, Inserm, CHU Lille, U1286—INFINITE—Lille Inflammation Research International Center, ICPAL, 3 Rue du Professeur Laguesse, 59000 Lille, France; natascha.leleu@univ-lille.fr (N.L.-C.); raphael.le-biannic@univ-lille.fr (R.L.B.); frederique.klupsch@univ-lille.fr (F.K.); 2 Univ. Lille, CHU Lille, ULR 7365—GRITA—Groupe de Recherche sur les Formes Injectables et les Technologies Associées, 59000 Lille, France; romain.regnault@univ-lille.fr (R.R.); mostafa.kouach@univ-lille.fr (M.K.); 3 LASIRE Laboratoire Avancé de Spectroscopie pour les Intéractions la Réactivité et l′Environnement, 59655 Villeneuve d′Ascq, France; hania.ahouari@univ-lille.fr (H.A.); herve.vezin@univ-lille.fr (H.V.); 4 Univ. Lille, FR 2638—IMEC—Institut Michel-Eugène Chevreul, 59655 Lille, France; 5 Univ. Lille, CHU Lille, CNRS, Inserm, UMR9020—UMR1277—Canther—Cancer Heterogeneity, Plasticity and Resistance to Therapies, 59000 Lille, France; romain.magnez@inserm.fr (R.M.); xavier.thuru@inserm.fr (X.T.); 6 Oncowitan, Scientific Consulting Office, 59290 Lille, France

**Keywords:** aldehyde reactivity, antioxidant, cancer, drug adducts, edaravone, PD-L1, pyrazolone

## Abstract

Small molecules targeting the PD-1/PD-L1 checkpoint are actively searched to complement the anticancer arsenal. Different molecular scaffolds have been reported, including phenyl-pyrazolone derivatives which potently inhibit binding of PD-L1 to PD-1. These molecules are structurally close to antioxidant drug edaravone (EDA) used to treat amyotrophic lateral sclerosis. For this reason, we investigated the capacity of five PD-L1-binding phenyl-pyrazolone compounds (**1**–**5**) to scavenge the formation of oxygen free radicals using electron spin resonance spectroscopy with DPPH/DMPO probes. In addition, the reactivity of the compounds toward the oxidized base 5-formyluracil (5fU) was assessed using chromatography coupled to mass spectrometry and photodiode array detectors. The data revealed that the phenyl-pyrazolone derivatives display antioxidant properties and exhibit a variable reactivity toward 5fU. Compound **2** with a *N*-dichlorophenyl-pyrazolone moiety cumulates the three properties, being a potent PD-L1 binder, a robust antioxidant and an aldehyde-reactive compound. On the opposite, the adamantane derivative **5** is a potent PD-L1 binding with a reduced antioxidant potential and no aldehyde reactivity. The nature of the substituent on the phenyl-pyrazolone core modulates the antioxidant capacity and reactivity toward aromatic aldehydes. The molecular signature of the compound can be adapted at will, to confer additional properties to these PD-L1 binders.

## 1. Introduction

Monoclonal antibodies (mAbs) targeting the programmed cell death ligand 1 (PD-L1) or its receptor programmed cell death 1 (PD-1) are essential components of the anticancer arsenal. They are extensively used to treat a variety of cancers, such as (non-)small cell lung cancer, melanoma, renal cell cancer, head and neck cancer, and many other tumor indications. Since 2014, 10 mAbs targeting PD-1 and three targeting PD-L1 have been approved by the different health agencies worldwide [1]. Other anti-PD-(L)1 mono- or bi-specific mAbs are in clinical development, often in combination with chemotherapeutic drugs or radiotherapy [2,3].

Beyond mAbs, small molecules targeting the PD-1/PD-L1 checkpoint are being actively searched. They would permit to reach tumors not easily accessible to mAbs, such as brain tumors. They would offer opportunities for oral treatment, compared to injectable mAbs. Moreover, they would reduce the overall cost of treatments, compared to costly mAb-based therapies [4]. Many small molecules targeting PD-1 or PD-L1 have been designed, chiefly represented by the leading compounds from by Bristol-Myers Squibb (BMS), BMS-202 and BMS-1166 [5,6]. Most of these PD-L1-targeted small molecules contain a biphenyl core, and function by inducing PD-L1 dimerization [7,8,9]. Different structural archetypes have been reported, including pyrazolo-pyridine derivatives [10], phenylindoline derivatives [11], triazine-based molecules [12] and various biphenyl compounds [13,14]. Potent compounds have been designed, with nanomolar affinities toward PD-L1, but only a very few have reached phase 1 clinical development [4,15].

**Figure 1:**
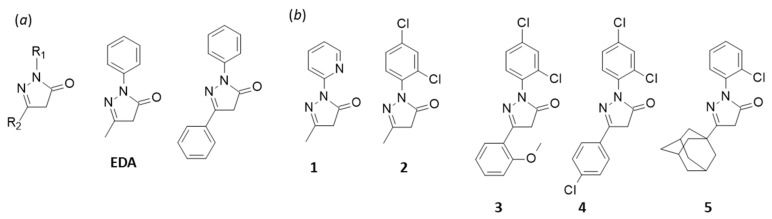
(**a**) Structure of edaravone (EDA), the pyrazolone central core and the phenyl-pyrazolone-phenyl unit found in our PD-L1 binders [17]. (**b**) Structure of the compounds (**1**–**5**) investigated.

In this context, we have recently reported the design of a novel series of PD-L1-targeted small molecules comprising a pyrazolone core scaffold. A hundred compounds have been synthesized and tested, leading to the identification of diverse PD-L1 binders [16]. Recently, a first series of 40 compounds have been reported, including several highly compounds with a nanomolar affinity for human PD-L1 and cell growth inhibitory properties [17]. Most of these molecules have in common an unfused bicyclic phenyl-pyrazolone or a tricyclic phenyl-pyrazolone-phenyl central skeleton (Figure 1a). Structurally, these PD-L1 binders are reminiscent to the drug edaravone (1-phenyl-3-methyl-5-pyrazolone) which is used to treat amyotrophic lateral sclerosis (ALS) and acute ischaemic stroke [18,19].

Edaravone (Radicut^®^, here designated EDA) is a cell protective agent, capable of reducing oxidative damages in various diseases, especially neurodegenerative diseases [20,21]. It is a potent antioxidant compound, acting as a scavenger for a large variety of oxygen free radicals [22,23]. In addition, EDA is a reactive molecule capable of forming covalent adducts with aldehydes. The drug has been shown to form stable adduct with pterin derivatives, such as 6-formylpterin, and other aldehydes [24,25,26,27,28]. By analogy, we reasoned that our phenyl-pyrazolone-based PD-L1 binders may display also antioxidant properties and a reactivity toward aldehydes. These two aspects have been investigated and the results are reported here.

We selected five compounds (**1**–**5**) among our best PD-L1 binders (Figure 1b). These five compounds bind to PD-L1 with nanomolar affinities (Table 1), and are able to induce PD-L1 dimerization. As recently described, compounds **1** and **5** have the capacity to disrupt PD-1/PD-L1 interaction and to inhibit the recruitment protein SHP2 (Src homology region 2 domain-containing phosphatase) to PD-1 [17]. In addition to the pyridine-type compound **1** and the adamantane-containing compound **5**, we selected three other compounds with a 2,4-dichlorophenyl unit at R_1_ but with a different R_2_ substituent: a methyl group (**2**), a 2-methoxyphenyl group (**3**) or a 3-chlorophenyl group (**4**). The five compounds were tested for their antioxidant capacity using two complementary electron spin resonance (EPR)-based assays. Their capacity to react with an aldehyde compound (5-formyluracil (5fU)) was investigated using analytical methods (liquid chromatography coupled to both mass spectrometry and DAD detectors). The results demonstrate that the compounds effectively present marked antioxidant properties and a reactivity toward aldehyde, in addition to their targeting of PD-L1. The levels of radical scavenging and covalent reaction with 5fU vary significantly from one compound to another. This important discovery will help to select and design the best drug candidates for subsequent studies in this phenyl-pyrazolone series.

## 2. Results

### 2.1. Antioxidant Activity

The antioxidant potency of the compounds was evaluated using two complementary EPR-based assays: a free radical scavenging test with 1,1-diphenyl-2-picrylhydrazyl (DPPH) and a spin-trapping method with 5,5-dimethyl-1-pyrroline *N*-oxide (DMPO) to trace the formation of hydroxyl radical in the presence of iron and hydrogen peroxide (Fenton reaction). The two sets of experiments are be discussed in turn.

#### 2.1.1. DPPH Assay

**Figure 2:**
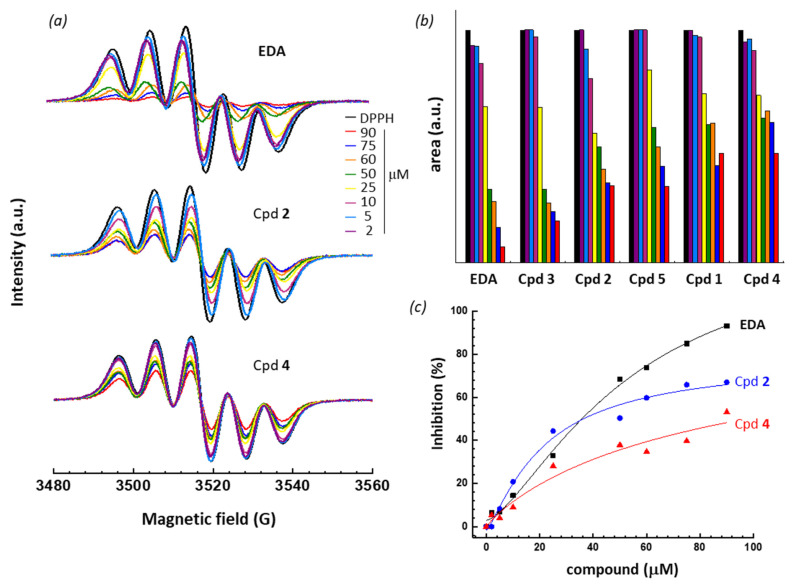
(**a**) EPR spectra of DPPH (100 μM) in the presence of increasing amounts of the indicated compound. (**b**) Comparison of the DPPH radical scavenging activity of the compounds. DPPH was used at 100 μM and the concentration of the test compound varied from 2 to 90 μM, as indicated. (**c**) Dose-dependent antioxidant activities of compounds EDA, **2** and **4**. Inhibition (%) of the DPPH signal intensity as a function of the compound concentration. These plots were used to determine EC_50_ values (Table 2). The color code is identical for panels 2a and 2b.

The antioxidant activity of the compounds was characterized for their free radical scavenging activities using DPPH (100 μM), chosen as a model radical. In this case, we monitor the decay in the DPPH EPR signal intensity which occurs when EPR-silent diamagnetic hydrazine counterparts are formed from DPPH. The first line DPPH signal intensity is then recorded in the presence of increasing amount of the test compound (2–90 μM). Representative dose-dependent variations of the EPR signal are shown in Figure 2a, for compounds EDA, **2** and **4**. They all show the characteristic five-line EPR spectrum of DPPH, due to the hyperfine coupling with two equivalent nitrogens, and for which the intensity is proportional to the DPPH concentration, and its decay in the presence of an antioxidant. With each compound, we observed a DPPH radical transformation into the corresponding diamagnetic derivative, indicating that all compounds have antioxidant properties. From these curves, the intensity of the EPR signal can be measured and its decay quantified (Figure 2b). It is immediately apparent that EDA displays a prominent antioxidant activity, with a strong capacity to reduce the intensity of the EPR DPPH signal, whereas compound **4** is less efficient.

A quantitative evaluation of the antioxidant effect can be made through the calculation of the concentration of compound required to reduce the EPR signal by 50% (EC_50_). For each compound, the percentage of inhibition of the EPR signal was plotted as a function of the compound concentration, and the fitted curves used to determine EC_50_ values. Representative plots are presented for compounds EDA, **2** and **4** (Figure 2c) and the EC_50_ values are indicated in Table 2. The same procedure has been used to evaluate various types of antioxidant compounds [29]. The results indicate that three compounds display approximately the same antioxidant capacity, EDA, **2** and **3**, with EC_50_ values in the 34–36 μM range. The three other compounds display a lower antioxidant capacity, with the bis-phenyl-pyrazolone compound **4** being the less efficient in the series with an EC_50_ value of 97 μM range. In this test, the compounds rank in the order EDA, **2**, **3** > **5** > **1** > **4**.

#### 2.1.2. DMPO Assay

**Figure 3:**
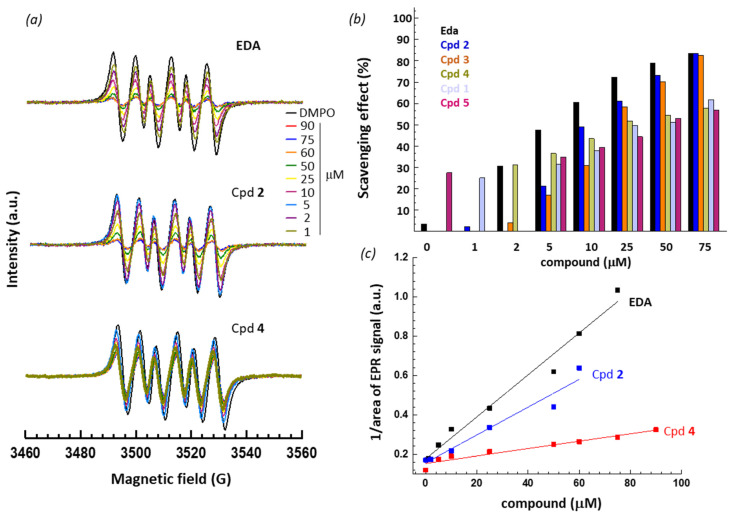
(**a**) EPR spectra of DMPO/^•^OCH_3_ radical in the presence of increasing amounts of the indicated compound. (**b**) Comparison of the scavenging effect (%) of the compounds. DMPO was used at 1 or 2 mM and the concentration of the test compound varied from 1 to 75 μM, as indicated. The scavenging effect (%) was calculated from the ratio [area(DMPO/^•^OCH_3_) − area(DMPO/^•^OCH_3_ + test compound)]/area(DMPO/^•^OCH_3_). (**c**) Dose-response curve to compare the antioxidant capacity of compounds EDA, **2** and **4**. These plots were used to determine the reaction rate constant (k_r_) (Table 1). The scavenging effect (%) is defined by the same ratio as mentioned above, as defined in the literature [29,30]. The goodness-of-fit values (r^2^ calculated after linear regression) ranged from 0.960 to 0.999.

In this case, we monitor the formation of an oxygen centered radical generated in the presence of the spin trapping agent DMPO under Fenton conditions (hydrogen peroxide plus ferrous ions). For practical reasons (compound solubility), experiments were carried out using methanol as a solvent and therefore, the methoxy radical is monitored. A typical six-line EPR signal is recorded, corresponding to the DMPO/^•^OCH_3_ radical, with the characteristic hyperfine coupling constants a_N_ = 13.9 G and a_H_ = 8.3 G. Here again, we can measure the decay of the EPR signal in the presence of the different antioxidant compounds and compare their relative capacity to reduce the signal. Typical concentration-dependent response curves are presented in Figure 3a for compounds EDA, **2** and **4**. All six compounds can induce a decrease of the EPR signal but with various degrees of efficacy (Figure 3b). Here again, the results were quantified through the measure of the area of the EPR signal as a function of the compound concentration, to allow the calculation of the reaction constants for each compound. Unsurprisingly, we observed that EDA exhibited a strong capacity to quench DMPO-trapped ^•^OCH_3_ radicals and the other test compounds revealed various degrees of efficacy. Proper dose-response curves were constructed and fitted to determine the reaction rate constant (k_r_) for each compound, as shown in Figure 3c and the k_r_ values are collated in Table 2.

There is no doubt that all compounds exhibit a marked antioxidant capacity. In this case, EDA appeared the best antioxidant in the series and the other compounds ranked in the order: EDA > **2** > **3** > **1** > **5** > **4**. The correlation between the DPPH and DMPO data is satisfactory, as in both cases we could rank the compounds in two groups. There are three potent antioxidant compounds: EDA, compounds **2** and **3**, and three less efficient compounds, **1**/**5** and the least efficient **4**. This latter molecule displays a much weaker capacity to quench oxygen-based radicals compared to **2** for example. However, it is worth to mention that in all cases the calculated reaction rate constants are in the same order of magnitude (k_r_ = 10^11^ M^−1^ s^−1^) as those measured with well-established compounds such as vitamins C (ascorbic acid) and E (α-tocopherol). The value measured with EDA is close to that reported in the literature [31], thus validating our measurements. Altogether, the DPPH and DMPO assays concur to show that the compounds present marked antioxidant effects, and the antioxidant capacity can be tuned through appropriate substitutions of the phenyl-pyrazolone core. A highly potent antioxidant agent, such as **2** and **3**, can be selected, or conversely it is possible to select a compound with a reduced antioxidant effect, such as **4**.

### 2.2. Reactivity toward Aromatic Aldehydes

The test compounds all possess a pyrazolone unit potentially reactive toward aldehydes, as previously observed with EDA [27,28]. The reactivity of the compounds toward an aldehydic compound was assessed using analytical methods. As a model aldehyde with a potential biological relevance, we chose 5-formyluracil (5fU), which can be formed in cells upon oxidation of the thymine methyl group. This modified base is present (at a low content) in cells and represents an important modification in genomic DNA [34,35].

#### 2.2.1. Adduct Formation in the Presence of 5-Formyluracil (5fU) (HRMS Analyses)

**Figure 4:**
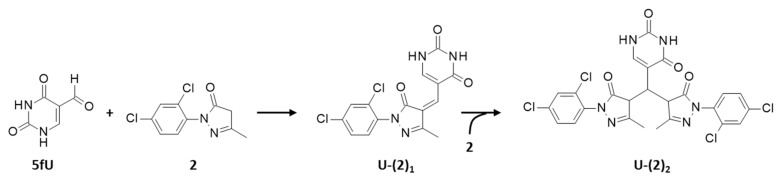
Reaction scheme for the formation of mono- and bis-adducts in the presence of compound **2** and 5-formyluracil (5fU).

To begin with, each compound was incubated with an equimolar amount of 5fU for 180 min at 37 °C in a mixture of methanol and ammonium formate buffer 5 mM, pH 4.5 (40:60, *v*:*v*). The compounds were tested at 0.5 mM, except compound **4** used at 0.1 mM due to its limited solubility in the aqueous medium. In each case, the reaction mixture was introduced into the mass spectrometry equipment after a dilution in methanol to achieve a final concentration of 10 pmol/µL. The formation of drug-uracil adducts was monitored and unambiguously characterized by HRMS. The data reported in Table 3 clearly indicate that, as expected, EDA forms mono- and bis-adducts in the presence of the aldehyde compound. Similarly, mono- and bis-adducts were detected in the presence of four compounds: **1**, **2**, **3** and **4**. In sharp contrast, compound **5** provided no adduct formation, even when tested at lower concentrations. There was no solubility issue with compound. The lack of reactivity is attributable to its adamantane unit which deactivates the adjacent reactive position or produces a steric hindrance. Otherwise, the other four compounds can react easily with 5fU to generate mono- and bis-adducts, as represented in Figure 4 with compound **2**. In all cases, the observed molar mass and ring double bound value (RDB) value for the different species coincide perfectly with the expected mass (Δppm < 5 ppm) and RDB value (Table 3).

#### 2.2.2. Separation and Characterization of Covalent Uracil-Drug Adducts (LC-HRMS Analysis)

**Figure 5:**
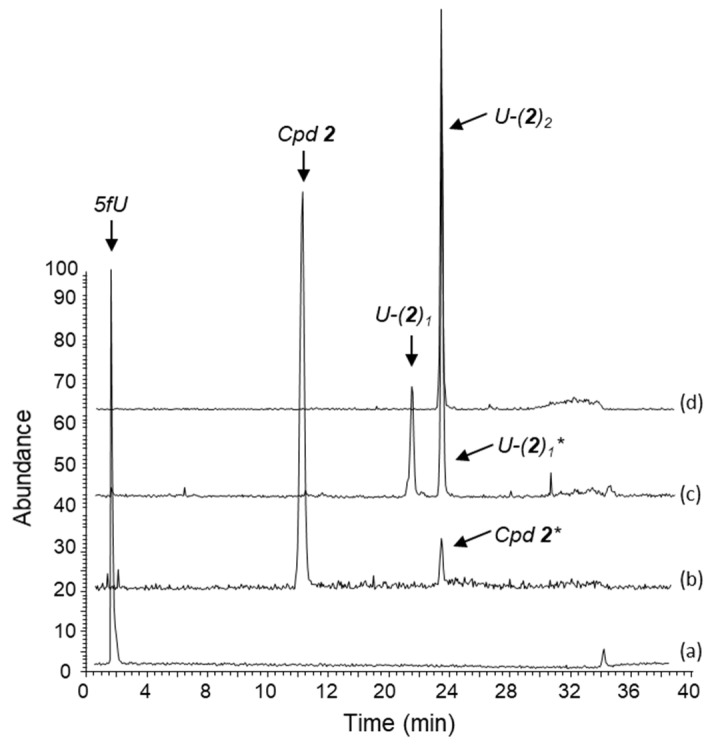
LC-HRMS analysis in negative mode [M-H]^-^ of mixture obtained upon reaction of the two compounds **2** and 5-formyluracil (5fU) (0.5 mM each), at 37 °C for 180 min in a methanol:ammonium formate buffer 5 mM, pH 4.5 mixture (40:60, *v*:*v*). The mass/charge ratio were recorded as follows: (a) 138.50 < m/z < 139.50 corresponding to 5-fU with a retention time (rt) of 1.20 min, (b) 240.50 < m/z < 241.50 corresponding to compound **2** with a rt of 14.43 min, (c) 362.50 < m/z < 363.50 corresponding to the expected mono adduct U-(**2**)_1_ with a rt of 21.86 min, and (d) 604.50 < m/z < 605.50 corresponding to the bis-adduct U-(**2**)_2_ with a rt of 23.94 min. In panel (c) and in panel (b), an additional signal with a rt of 23.93 min are observed. Their correspond to the in source-fragmentation (ISF) of the bis-adduct during the electrospray ionization (ESI) process. The pics marked * (at rt = 23.90 min) refer to the ISF of U-(**2**)_2_ to generate both ion fragments analogous to U-(**2**)_1_ and the starting compound **2**.

To further characterize the reaction products, we performed a LC-HRMS analysis with each compound. A typical profile obtained with compound **2** is presented in Figure 5. The aldehyde reactant (trace a) has a shorter retention time (rt = 1.20 min) than the pyrazolone compound **2** (rt = 14.43 min). Traces c and d correspond to the record of the mono-adduct (362.50 < m/z < 363.50) and bis-adduct (604.50 < m/z < 605.50), with retention times (rt) of 21.86 min and 23.94 min, respectively. In trace c, we observed two clear signals (rt = 21.86 and 23.93 min), the first is the ion U-(2)_1_ and the second is also the ion U-(2)_1_ (marked U-(2)_1_* in Figure 5) due to the in source-fragmentation (ISF) of the bis-adduct U-(2)_2_. ISF is also at the origin of the small peak in trace b corresponding to the release of the compound **2** (marked 2* in Figure 5). ISF is a naturally occurring phenomenon during electrospray ionization (ESI) analysis. It is influenced by different parameters such as capillary voltage, end plate offset and ion energy [36]. An ESI fragmentation was observed with the non-conjugated bis-adduct, not seen with the conjugated mono-adduct probably more chemically stable due to the double bond between pyrazolone and pyrimidin rings. This HRMS characterization of the reaction products demonstrates unambiguously the formation of covalent uracil adducts with the different compounds.

#### 2.2.3. Kinetic of Formation of Mono- and Bi-Adducts

A chromatographic separation of the reaction products has been performed by liquid chromatography coupled to an absorption detection at 254 nm or 360 nm. The two wavelengths are useful to facilitate the detection of the products. As illustrated in Figure 4 with compound **1**, the first covalent reaction of the product with 5fU leads to the formation of a mono-adduct U-(**1**)_1_ presenting a conjugated double bond system between the pyrazolone and uracil units. This mono-adduct can be easily detected by absorption at 360 nm. Then, as the reaction proceeds further, a bis-adduct U-(**1**)_2_ is formed and the conjugation between the two pyrazolones and the uracil unit is lost. This bis-adduct is therefore better detected under an UV light (254 nm) than under visible radiation (360 nm). This was expected for a Knoevenagel-type condensation of an aromatic aldehyde. The nucleophilic addition of the aldehyde leads to the deprotonation of the pyrazolone methylene group, to form an extended conjugated system for the mono-adduct. In contrast, the subsequent condensation gives the bis-adduct characterized by a non-conjugated system.

**Figure 6:**
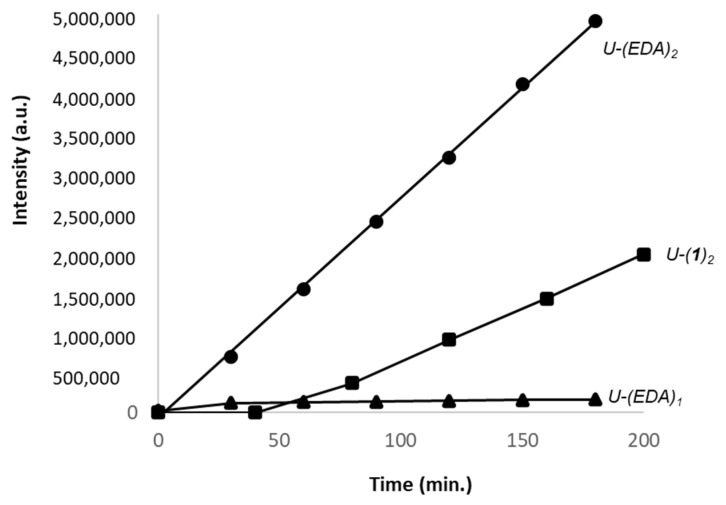
Kinetic of formation of the mono-adduct U-(EDA)_1_ (triangles) and bis-adduct U-(EDA)_2_ (circles) and U-(**1**)_2_ (squares) formed with EDA or **1** in the presence of 5fU. Due to the weak signal intensity of the mono-adduct U-(**2**)_1_ at 254 nm, it is not represented here. EDA or compound **1** (0.5 mM) was incubated with 5fU (0.5 mM) at 37 °C for 180 min in a methanol:ammonium formate buffer 5 mM, pH 4.5 mixture (40:60, *v*:*v*). The reaction products were analyzed by HPLC-DAD and the surfaces of the pics corresponding to the mono- and bis-adducts were recorded at 254 nm.

For each compound, we analyzed the reaction products formed over a period of 180 min and quantified the formation of mono- and bis-adducts. An equimolar amount of 5fU and the test compound (0.5 mM each) was reacted at 37 °C in the mixture containing methanol and ammonium formate buffer 5 mM, pH 4.5 (40:60). Typical plots obtained with compounds EDA and **1** are shown in Figure 6. The bis-adduct is formed at a considerably higher rate compared to the mono-adduct. The variation of the surface of U(EDA)_2_ vs. time allows determination of the initial reaction rate from the slope of the linear variation expressed in AU/min.

**Figure 7:**
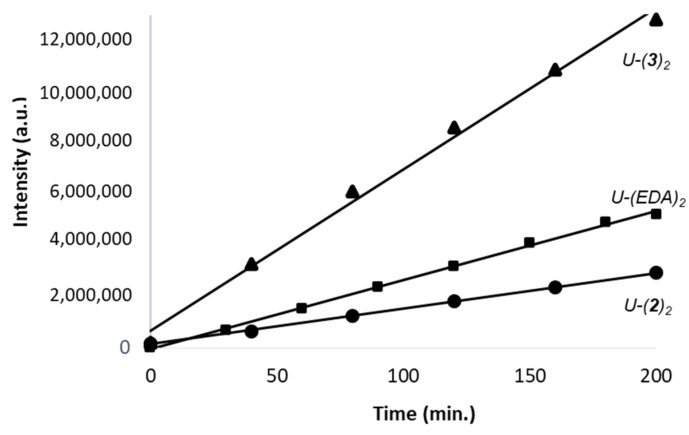
Comparison of the time-dependent formation of the bis-adduct for compounds **2** (circles) and **3** (triangles) in the presence of 5fU, vs. EDA (squares). Each compound (0.5 mM) was incubated with 5fU (0.5 mM) at 37 °C for 200 min in a methanol:water, ammonium formate buffer, 5 mM, pH 4.5 mixture (40:60, *v*:*v*). The reaction products were analyzed by HPLC-DAD and the surfaces of the pics corresponding to the bis-adducts were recorded at 254 nm.

A similar analysis was performed with each compound. In each case, the reaction proceeds rapidly toward the formation of bis-adduct compared to the mono-adduct. The bis-adduct is always the major species formed in the presence of 5fU. Typical plots obtained with compounds **2**, **3** and EDA are presented in Figure 7. The rates of formation (slopes of the linear variation) of the bis-adducts can be compared.

We observed that the nature of the phenyl substituent on the pyrazolone unit can modulate the level of reaction with 5fU. Compared to EDA, the two compounds **1** and **2** exhibited a similar lower reactivity toward 5fU. The calculated reactivity coefficient was 0.2 compared to 1 with EDA (the coefficient is the ratio of the calculated reaction rate, compound/EDA). Compound **4** was even less reactive than EDA at 0.1 mM, with a reactivity coefficient of 0.33. In sharp contrast, compound **3** proved to be more reactive than EDA, with a calculated reactivity coefficient of 5.2. The high reactivity of this compound toward 5fU compared to EDA can be seen most clearly in Figure 7, with a rate of formation of the corresponding bis-adduct significantly superior to that of EDA. There is no doubt that the nature of the substituents on the phenyl-pyrazolone unit significantly influences the aldehyde reactivity of the compound. The replacement of the phenyl group of EDA with a pyridine ring (**1**) reduces the nucleophilic potential of the molecule, as does the incorporation of two chlorine atoms on the phenyl moiety (**2**). The incorporation of a para-chlorophenyl moiety further reduces the reactivity (**4**) but, on the opposite, the inclusion of an ortho-methoxyphenyl group (**3**) enhances markedly the aldehyde reactivity of the compound. The o-OCH_3_ substituent certainly changes the local electron-density distribution, so as to enhance the reactivity at the proximal methylene unit on the pyrazolone ring. The o-OCH_3_ group likely donates electrons which increase the energy density at the ionizable group. We can therefore modulate the aldehyde reactivity of the compounds at will, from a non-reactive molecule (**5**) or weakly reactive compounds (**1**,**2**,**4**) to a highly reactive compound (**3**). The modularity of the system is interesting.

## 3. Discussion

The structural analogy between the phenyl-pyrazolones targeting PD-L1 recently developed and the anti-ALS drug edaravone prompted us to investigate the reactivity of five compounds (selected among potent PD-L1 binders) toward reactive oxygen species and an aromatic aldehyde. EDA is known for a long time as a potent antioxidant drug, capable of scavenging a multitude of oxygen and nitrogen radicals in living systems [22,31,37]. Unsurprisingly, the different analogues studied here share this capacity to scavenge oxygen radicals, as judged from the EPR experiments using the DPPH/DMPO probes. The phenyl-pyrazolone skeleton of EDA can be modulated without abolishing the antioxidant potential. Among the six products tested here, EDA is the best antioxidant and the various structural changes do not drastically reduce this potential. This observation was somewhat expected, at least for the di-aromatic phenyl-pyrazolones. The pyridine derivative **1** has been described 11 years ago and shown to behave as a potent antioxidant [38]. Similarly, a chlorinated analogue of EDA has been shown previously to maintain a significant antioxidant capacity, just slightly inferior to that of EDA [38]. It is therefore not entirely surprising to observe that our phenyl-pyrazolone derivatives behave as antioxidants, including the unfused tricyclic compounds such as **3**/**5**. In contrast with EDA (which is totally inactive toward PD-L1), our compounds are PD-L1 binders coupled with antioxidant properties.

The antioxidant effect of the compounds can have an effect on the expression of PD-L1 at the surface of cancer cells [39]. We consider that the development of a PD-L1 inhibitor with marked antioxidant properties can be beneficial. It has been shown that antioxidants can enhance the efficacy of immunotherapy via different mechanisms: (i) via a down-regulation of PD-L1 expression in cancer cells, as observed with the antioxidant natural products such as cardamonin, nobiletin, sesamin and hesperetin [40,41,42,43], (ii) via an upregulation of major histocompatibility complex and surface activation molecules in dendritic cells, as reported with the antioxidant polysaccharide fucoidan [44], (iii) via the stimulation of TET2 enzyme leading to an increased intratumoral infiltration of T cells and the expression of cytokines and chemokines, as found recently with the prototypical antioxidant ascorbic acid [45], or (iv) via other mechanisms implicating the HIF1α/STAT3 pathway for example [46]. Ascorbic acid (vitamin C) has been shown to empower cancer immunotherapy through its pro-oxidant potential, based on its capacity to modulate epigenetic factors and to regulate expression of different cytokines involved in the immune response [47,48]. There are good evidence showing that redox-active treatments can reduce antitumor immunity in part via modulation of the PD-1/PD-L1 checkpoint [49]. Redox regulation with antioxidant compounds can be used to abrogate PD-L1 expression cancer cells [50]. For this reason, we believe that a phenyl-pyrazolone derivative directed against PD-L1 and exhibiting a marked antioxidant effect could present strong immuno-modulatory and antitumor capacities in vivo.

The reactivity toward a model aldehyde, 5-formyluracil, is much more sensitive to the compound structure than the antioxidant potential. Compared to EDA, there is a possibility to completely abrogate this reactivity or to promote it. The adamantane compound **5** has lost the aldehyde reactivity. On the opposite, the tricyclic compound **3** with a 2-methoxy-phenyl substituent displays an enhanced reactivity toward formyl uracil. Therefore, this reactivity can be tuned on demand. The biological significance of this aldehyde reactivity, if any, is not known at present. It may be a source of unwanted reactions with endogenous aromatic aldehydes (5-formyluracil, 5-formylcytosine, 6-formylpterin, and others) or it may be a beneficial effect to modulate the epigenetic machinery and the functioning of TET (10-11 translocation) enzymes implicated in methyl-cytosine oxidation. We will investigate these aspects in the near future. Nevertheless, it is important to realize that the reactivity can be modulated, without comprising the PD-L1 binding capacity. Newer derivatives are currently being synthesized, with a modulated pyrazolone scaffold to reinforce the metabolic stability of these compounds and delineate further the structure-function in the series.

## 4. Materials and Methods

### 4.1. Chemicals

Pyrazolones were synthesized by the Knorr reaction, a condensation between a hydrazine and a β-keto ester [17]. The detailed procedure for the microwave-assisted synthesis of compounds 1 has been reported recently [17]. Briefly, the corresponding β-keto ester (1 eq.) and hydrazine (1.5 eq.) were dissolved in glacial acetic acid (6 M). The mixture was subjected to microwave irradiations for 2.5–5 min at 130 °C and 150 W. The solvent was evaporated under reduced pressure. Compound 1 was purified by flash chromatography using a gradient of eluent cyclohexane:ethyl acetate from (1:0) to (8:2). A similar procedure has been adapted for the synthesis of compound 2. The complete characterization of 2 has been reported [51]. The synthesis of the pyrazolone derivatives 3, 4 and 5 has been described in a patent [16]. To a solution of the corresponding β-cetoester (1 mmol, 1 eq.) in acetic acid (5 mL) were added sodium acetate (0.5 eq.) and hydrazine (1 eq.). The reaction mixture was stirred for 48 h at room temperature. After reaction, the solvent was evaporated under reduced pressure. The residue was dissolved in ethyl actetate (10 mL) and washed with water (10 mL) and brine (10 mL). The organic layer was dried over MgSO_4_ and evaporated under reduced pressure. Compounds 3 and 5 were purified by chromatography on silica gel using cyclohexane:ethyl acetate (8:2 and 7:3, respectively) as eluent. Compounds 4 and 5 were recrystallized in acetonitrile. Compound 3 was recrystallized in absolute ethanol. Complete characterizations of compounds 3, 4 and 5 are described in the patent [16].

### 4.2. PD-L1 Binding and PD-L1-Dependent Activity

The capacity of the compounds to bind to recombinant human PD-L1 was assessed by microscale thermophoresis (MST), as previously described [52]. Their capacity to interfere with PD-L1 activity in cells and to modulate cellular proliferation has been recently described [17].

### 4.3. EPR Measurements

CW-EPR experiments were performed with an X-band Bruker Elexsys E500 spectrometer (Bruker BioSpin GmbH, Rheinstetten, Germany) operating at 9.86 GHz. CW spectra were recorded at room temperature with a microwave power of 5.024–10.02 mW and a modulation amplitude of 1–2 G that assumes non-saturation conditions. The antioxidant activity was monitored using DPPH (1,1-diphenyl-2-picrylhydrazyl) at a final concentration of 100 µM as free radical scavenging molecule and DMPO (5,5-dimethyl-1-pyrroline *N*-oxide) as spin trap agent for the spin trapping experiments. The DMPO solution was freshly distillated prior to use at a final concentration of 1 mM or 2 mM. The solution mixture was filled into a glass capillary of 50 μL, which was in turn, placed in 4 mm quartz EPR tubes prior to analysis.

The percent inhibition of DPPH was calculated according to the following equation: I (%) = ((I_0_ − I)/I_0_) ∗ 100 where I_0_ is the area of the EPR spectrum of DPPH (control sample), and I is the area of the EPR spectrum of DPPH with antioxidant compound. The rate constant for the reaction of antioxidant compounds with ^•^OCH_3_ is expressed by the following equation: 1/R_a_ = 1/R_f_ + (k_r_ [AH]/(k_a_ [DMPO] × R_f_). R_f_, and R_a_ indicate EPR signal area (double integration of EPR signal) of ^•^OCH_3_, DMPO/^•^OCH_3_, respectively [31,32]. k_a_ and [DMPO] are constants, and a plot of 1/R_a_ vs. [AH] (concentration of antioxidant compound) gives a straight line with 1/R_f_ as an intercept and k_r_/(k_a_[DMPO] R_f_) as the slope. In these circumstances, the rate constant for the reaction of ^•^OCH_3_ with antioxidant compounds (k_r_) can be expressed by the following equation: k_r_ = (k_a_ [DMPO] × slope)/interception.

### 4.4. Aldehyde Reactivity Measurements

#### 4.4.1. High Resolution Mass Spectrometry (HRMS) and LC-HRMS Methods

HRMS spectra were recorded using an Exactive mass spectrometer (Thermo Fisher Scientific, San Jose, CA, USA) equipped with a heated electrospray ionisation probe (HESI -II). Each mass analyzer was calibrated with Pierce^®^ESI positive and negative ion calibration solutions each week (Thermo Fisher Scientific). Optimization of voltages, gas values, and temperatures applied for ion transfer and ionization was performed in negative mode. The tuning parameters were optimized separately with reaction mixture obtained from reaction of EDA with the aldehyde. This was carried out by infusing individual solutions of EDA or the aldehyde at 10 pmol/µL and then the diluted reaction mixtures (1/50 in MeOH) after 24 h reaction, at a flow rate of 10 µL/min into the mobile phase (0.4 mL/min) using a T connection. MS parameters were set as follows: sheath gas flow rate 45; auxiliary gas flow rate 10 arbitrary units (nitrogen was used as auxiliary and sheath gas); spray voltage −3.50 kV; capillary temperature 320 °C; auxiliary gas heater temperature 320 °C and S-lens RF level 50. The ions between m/z 150–1000 were scanned in ultrahigh-resolution mode of instrument.

The identification of compounds, EDA or each aldehyde, and reaction products was performed by HRMS data as elementary analysis parameters (m/z values, Ring Double Bond values and mass accuracies lower than 5 ppm). For the reactivity study of EDA or compounds **1**–**5** with 5-formyluracil (5fU), analyses of solutions (prepared as described above) were further performed using an UHPLC Accela system coupled to the Exactive mass spectrometer with the same detector parameters as described above. Separations were carried out on a reversed-phase Biozen XB-C18 (100 × 2.1 mm i.d., 2.6 µm) column (Phenomenex, Le Pecq, France). A gradient separation mode was used to separate the analytes, using H_2_O (A) and methanol (B), both containing ammonium formate 5 mM and formic acid 0.02% (*v*:*v*). The elution gradient performed at 0.25 mL/min was the following: (i) at the starting condition, the proportion was 40:60 MeOH:H_2_O (*v*:*v*) up to 1 min; (ii) the proportion of methanol was increased from 40 to 60 % in 20 min and then was kept constant for one minute; (iii) the proportion of methanol was increased to 70 % in one minute and remained constant for one minute before returning to the initial conditions. The column was thermostated at 30 °C and the injection volume was 5 µL. The software Xcalibur was used for the LC-HRMS system control, data acquisition and processing.

#### 4.4.2. HPLC-DAD Method

Chromatographic analyses were performed using an HPLC Alliance system (Waters, Milford, MA, USA) equipped with a gradient quaternary pump, an on-line degasser apparatus, an autosampler and a 2996-photodiode array detector. Data were collected and processed on a computer running with Empower software (version 3) from Waters Corporation (Milford, CT, USA). Separations were carried out with the same stationary and mobile phases described in Section 2.2.3 except for the flow rate fixed at 0.20 mL/min and the 20 μL injection volume. The autosampler temperature was 37 °C. Detection was performed at 254 nm or 360 nm. Serial dilutions of EDA, the test compounds and 5-formyluracil stock solutions (10–100 mM) were prepared using DMSO, prior to dilutions in the mixture of methanol and ammonium formate buffer 5 mM, pH 4.5.

## 5. Conclusions

Phenyl-pyrazolone derivatives capable of potently inhibiting the interaction of the PD-L1 ligand with its receptor PD-1 to block cancer cell proliferation have been discovered. In addition to interacting with PD-L1, these molecules exhibit significant antioxidant effects, characterized by the scavenging of oxygen radicals. This property is reminiscent of the antioxidant property of the structurally related drug edaravone. Moreover, depending on the nature of the substituent on the phenyl-pyrazolone scaffold, the compounds can react with aromatic aldehydes such as 5-formyluracil. This property can be modulated, either suppressed or amplified, depending on the substituents on the phenyl-pyrazolone core. Based on these properties, the design of novel PD-L1-targeted small molecules is pursued. 

## Figures and Tables

**Table 1:** PD-L1 binding and regulatory properties of the test compounds.

Compounds	PD-L1 Binding	Bioactivity	Proliferation
	(K_D_, nM) ^a^	(IC_50_, nM) ^b^	(IC_50_, nM) ^c^
**1**	77 ± 7	92 ± 9	102 ± 8
**2**	34 ± 3	23 ± 3	53 ± 11
**3**	45 ± 7	44 ± 9	79 ± 7
**4**	7 ± 3	3 ± 2	124 ± 7
**5**	12 ± 2	23 ± 6	57 ± 9
BMS-202	ND	124 ± 12	53 ± 17
Nivolumab	ND	ND	58 ± 3

^a^ Affinity for PD-L1 measured by microscale thermophoresis (MST). ^b^ Capacity of the compound to disrupt the PD-L1/PD-1 interaction in cells, measured by a fluorescence resonance energy transfer (FRET) assay. ^c^ Capacity of the compound to reactivate proliferation of CTLL-2 cells. ND = not determined.

**Table 2:** Antioxidant properties of the test compounds.

Cpd	DMPO		DPPH
	k_r_ (10^11^ M^−1^ s^−1^)	k_r_/k_a_	EC_50_ (μM)
EDA	2.59	60	35.81
**1**	1.27	30	70.58
**2**	1.93	45	36.46
**3**	1.88	44	34.02
**4**	1.04	24	97.58
**5**	1.25	29	59.74

DMPO was used at 1 or 2 mM. k_r_/k_a_ refers to the ratio of the reaction rate constant (k_r_) measured with the test compounds and the reaction rate constant (k_a_ DMPO-^•^OH) = 4.3 × 10^9^ M^−1^ s^−1^) considered for DMPO, as defined in the literature [32,33].

**Table 3:** HRMS data for the reaction of the studied products with 5-formyluracil (5fU).

Analyte [M-H]^−^	Formula	Ions m/z(Theorical)	Ions m/z(Observed)	Error Δppm	RDB Negative Mode
5fU	C_5_H_3_N_2_O_3_	139.022	139.013	−3.946	5.5
EDA	C_10_H_9_N_2_O	173.080	173.070	−2.713	7.5
U-(EDA)_1_	C_15_H_11_N_4_O_3_	295.090	295.082	−1.141	12.5
U-(EDA)_2_	C_25_H_21_N_6_O_4_	469.170	469.161	−1.960	18.5
Cpd **1**	C_9_H_8_N_3_O	174.075	174.066	−2.576	7.5
U-(**1**)_1_	C_14_H_10_N_5_O_3_	296.086	296.078	−0.627	12.5
U-(**1**)_2_	C_23_H_19_N_8_O_4_	471.160	471.152	−1.799	18.5
Cpd **2**	C_10_H_7_N_2_OCl_2_	241.001	240.993	−0.269	7.5
U-(**2**)_1_	C_15_H_9_N_4_O_3_Cl_2_	363.013	363.004	−0.639	12.5
U-(**2**)_2_	C_25_H_17_N_6_O_4_Cl_4_	605.014	605.005	−2.149	18.5
Cpd **3**	C_16_H_11_N_2_O_2_Cl_2_	333.028	333.019	−0.299	11.5
U-(**3**)_1_	C_15_H_9_N_4_O_3_Cl_2_	455.039	455.030	−1.421	16.5
U-(**3**)_2_	C_25_H_17_N_6_O_4_Cl_4_	789.067	789.056	−2.648	26.5
Cpd **4**	C_15_H_8_N_2_OCl_3_	336.977	336.969	−2.352	11.5
U-(**4**)_1_	C_20_H_10_N_4_O_3_Cl_3_	458.990	458.980	−3.507	16.5
U-(**4**)_2_	C_35_H_19_N_6_O_4_Cl_6_	796.68	796.956	−4.612	26.5
Cpd **5**	C_19_H_20_N_2_OCl	327.134	327.125	−1.704	10.5
U-(**5**)_1_	C_24_H_22_N_4_O_3_Cl	449.146	No reaction		
U-(**5**)_2_	C_43_H_43_N_6_O_4_Cl_2_	777.280	No reaction		

RDB, Ring Double Bound value. Δppm, delta ppm from the theoretical m/z values.

## Data Availability

Not applicable.
